# Pre- and Post-Zygotic TP53 De Novo Mutations in SHH-Medulloblastoma

**DOI:** 10.3390/cancers12092503

**Published:** 2020-09-03

**Authors:** Jacopo Azzollini, Elisabetta Schiavello, Francesca Romana Buttarelli, Carlo Alfredo Clerici, Laura Tizzoni, Giovanna De Vecchi, Fabio Capra, Federica Pisati, Veronica Biassoni, Letterio Runza, Giorgio Carrabba, Felice Giangaspero, Maura Massimino, Valeria Pensotti, Siranoush Manoukian

**Affiliations:** 1 Unit of Medical Genetics, Department of Medical Oncology and Hematology, Fondazione IRCCS Istituto Nazionale dei Tumori, 20133 Milano, Italy; Jacopo.Azzollini@istitutotumori.mi.it (J.A.); Siranoush.Manoukian@istitutotumori.mi.it (S.M.); 2 Pediatric Oncology Unit, Department of Medical Oncology and Hematology, Fondazione IRCCS Istituto Nazionale dei Tumori, 20133 Milano, Italy; elisabetta.schiavello@istitutotumori.mi.it (E.S.); veronica.biassoni@istitutotumori.mi.it (V.B.); 3 Department of Human Neurosciences, Sapienza University of Rome, 00185 Roma, Italy; francesca.buttarelli@uniroma1.it (F.R.B.); felice.giangaspero@uniroma1.it (F.G.); 4 Department of Oncology and Hemato-Oncology, University of Milan, 20122 Milan, Italy; carloalfredo.clerici@istitutotumori.mi.it; 5 Unit of Clinical Psychology, Fondazione IRCCS Istituto Nazionale dei Tumori, 20133 Milano, Italy; 6 Cancer Genetics Test Laboratory, Cogentech s.r.l. Società Benefit a Socio Unico, 20139 Milan, Italy; laura.tizzoni@cogentech.it (L.T.); giovanna.devecchi@cogentech.it (G.D.V.); fabio.capra@cogentech.it (F.C.); valeria.pensotti@cogentech.it (V.P.); 7 Histopathology Unit, Cogentech s.r.l. Società Benefit a Socio Unico, 20139 Milan, Italy; federica.pisati@cogentech.it; 8 Division of Pathology, Fondazione IRCCS Ca’ Granda, Ospedale Maggiore Policlinico, 20122 Milan, Italy; letterio.runza@policlinico.mi.it; 9 Division of Neurosurgery, Fondazione IRCCS Ca’ Granda Ospedale Maggiore Policlinico, 20122 Milan, Italy; giorgio.carrabba@policlinico.mi.it; 10 IRCCS Neuromed-Istituto Neurologico Mediterraneo Pozzilli, 86077 Pozzilli, Italy

**Keywords:** TP53, somatic mosaicism, medulloblastoma, de novo mutation, Li-Fraumeni syndrome

## Abstract

**Simple Summary:**

Medulloblastoma is the most common malignant brain tumor in children. In a subset of cases, a causal factor is a constitutive mutation of the TP53 gene, which may be inherited or arise for the first time in a patient (de novo). Using an immunohistochemistry assay as a screening tool, we selected patients suspected of harboring a TP53 mutation and offered genetic counseling and germline testing. Our study, which was the first to investigate the parental origin of TP53 mutations in medulloblastoma, allowed the identification of two additional cases with de novo mutations. Moreover, we demonstrated that in one patient the mutation originated at a post-zygotic stage, resulting in somatic mosaicism. These findings have important implications for genetic counseling since they highlight the occurrence of both pre- and post-zygotic TP53 de novo mutations in medulloblastoma, pointing out that in a specific subgroup of patients genetic testing should be offered regardless of family history.

**Abstract:**

Li-Fraumeni syndrome (LFS) is an autosomal dominant disorder caused by mutations in the *TP53* gene, predisposing to a wide spectrum of early-onset cancers, including brain tumors. In medulloblastoma patients, the role of TP53 has been extensively investigated, though the prevalence of de novo mutations has not been addressed. We characterized TP53 mutations in a monocentric cohort of consecutive Sonic Hedgehog (SHH)-activated medulloblastoma patients. Germline testing was offered based on tumor p53 immunostaining positivity. Among 24 patients, three (12.5%) showed tumor p53 overexpression, of whom two consented to undergo germline testing and resulted as carriers of TP53 mutations. In the first case, family history was uneventful and the mutation was not found in either of the parents. The second patient, with a family history suggestive of LFS, unexpectedly resulted as a carrier of the mosaic mutation c.742=/C>T p.(Arg248=/Trp). The allele frequency was 26% in normal tissues and 42–77% in tumor specimens. Loss of heterozygosity (LOH) in the tumor was also confirmed. Notably, the mosaic case has been in complete remission for more than one year, while the first patient, as most TP53-mutated medulloblastoma cases from other cohorts, showed a severe and rapidly progressive disease. Our study reported the first TP53 mosaic mutation in medulloblastoma patients and confirmed the importance of germline testing in p53 overexpressed SHH-medulloblastoma, regardless of family history.

## 1. Introduction

Li-Fraumeni syndrome (LFS; OMIM #151623) is a rare and heterogeneous genetic condition, characterized by an increased risk of developing a wide spectrum of tumors, including sarcomas, adrenocortical carcinoma (ACC), breast cancer, and brain tumors [1]. The *TP53* gene (chromosome location 17p13.1; OMIM #191170), encoding the ubiquitously expressed transcription factor p53, is the only LFS-associated gene known to date and it is also the most frequently somatically disrupted gene in sporadic cancers [2]. Heterozygous *TP53* germline pathogenic variants (mutations) are inherited in an autosomal dominant pattern and carriers typically display a significant personal and family history of early onset tumors [3].

However, the increasingly widespread use of next-generation sequencing (NGS) techniques has highlighted that, as for many other conditions, the rate of de novo *TP53* variants is substantially higher than previously expected, being currently estimated at about 15% of all detectable mutations [4].

Another related phenomenon, which emerged thanks to the NGS reading depth and has increasingly gained relevance, is the identification of de novo *TP53* mutations, occurring at a post-zygotic stage and leading to somatic mosaicism, in a considerable fraction of cancer-affected individuals. Although mosaicism is consistently identified following the detection of a lower allele frequency at germline testing, it might occur at discrete stages of post-zygotic development, with significantly different clinical implications.

Several studies pointed out that low allele frequencies (<25–35%) in blood from older patients might result from aberrant clonal expansion (ACE) events, mostly due to age- or therapy-related clonal hematopoiesis of indeterminate potential (CHIP) [5,6,7,8,9]. In the study from Weitzel and colleagues on 1454 adult patients tested with multigene panels including *TP53*, about 20% of the positive patients showed a low allele frequency, most of whom had the mutation confined to the blood [6].

On the other hand, the high sensitivity of NGS has also allowed a more efficient identification of young LFS patients with somatic mosaicism occurring at an early post-zygotic stage and thus, involving multiple tissues. In a large cohort of 328 unrelated LFS patients with *TP53* mutations at germline testing, somatic mosaicism was identified in 2.8% of cases (8/328) [4].

Childhood medulloblastoma is a tumor in the spectrum of LFS. Notably, somatic TP53 mutations in medulloblastoma are considered a crucial marker for molecular subgrouping and risk stratification, with important implications on prognosis and treatment [10,11,12,13]. A fast and reliable method to identify p53-defective tumors is p53 immunohistochemical (IHC) staining. This assay for the evaluation of p53 overexpression, which usually indicates the presence of gene alterations leading to impaired protein function and nuclear accumulation [14], showed a very high sensitivity (>90%) for the detection of TP53 somatic mutations in medulloblastoma and other malignancies [10,11,15]. As concerns the mutation detection rate, Tabori and colleagues reported that more than half (8/15) of p53 overexpressed medulloblastomas resulted TP53-mutated [11]. Although the global germline mutation prevalence in medulloblastoma is about 1% [13], a study showed that in nearly half (9/20) of patients with TP53-mutated medulloblastoma, the mutation was found to be germline [10]. However, data on the parental origin of germline mutations in large medulloblastoma cohorts are lacking and the prevalence of de novo mutations is not known.

In our study, we report on the molecular characterization of TP53 germline mutations in a monocentric cohort of patients affected with Sonic Hedgehog (SHH)-activated medulloblastoma and that resulted positive in p53 IHC staining.

## 2. Results

Since January 2015, a total of 24 medulloblastomas were classified as Sonic Hedgehog (SHH)-activated in the centrally reviewed histopathological examination. The p53 IHC assay identified three tumors (12.5%) with a positive staining, pointing out a putative underlying TP53 gene mutation. On the basis of this finding, the three patients and their parents were referred to genetic counseling for germline testing. One patient, a girl diagnosed with desmoplastic/nodular medulloblastoma at the age of 14 years, with no evidence of LFS-associated tumors in other family members up to the third degree of kinship, was not included in the study, since her parents did not consent to further genetic testing. In the other two cases, germline testing was carried out on both probands (Patient 1 and Patient 2) and their parents.

### 2.1. Patient 1

A 5-year-old girl with worsening headache and vomiting was admitted to a pediatric hospital for clinical and radiological evaluations. Brain CT scans and MRI revealed a left cerebellar lesion and hydrocephalous (Figure S1a). The patient underwent an emergency external cerebrospinal fluid (CSF) diversion followed by gross total tumor resection. Postoperative brain MRI showed a small tumor residue along the lateral margin of the surgical cavity (Figure S1b). No leptomeningeal dissemination in the spinal MRI and CSF evaluation was detected.

Centrally reviewed morphological and histological evaluation revealed a desmoplastic/nodular medulloblastoma, SHH-activated and p53 overexpressed (Figure S2).

The patient was treated with neoadjuvant chemotherapy consisting of one cycle of high dose methotrexate/vincristine and high dose cyclophosphamide/vincristine with autologous stem cells harvesting. Since local progression was observed at brain MRI evaluation, the intended myeloablative chemotherapy was replaced with hyperfractionated (46 fractions) accelerated craniospinal irradiation (31.2 Gy) with a boost dose to the tumor bed (28.6 Gy). Following radiotherapy, brain MRI showed a partial response, and adjuvant chemotherapy with cisplatin/vincristine/lomustine was administered for three cycles. Eleven months after surgery and during chemotherapy, a very aggressive local cerebellar progression was evidenced (Figure S1c) and the patient died shortly after.

**Figure 1:**
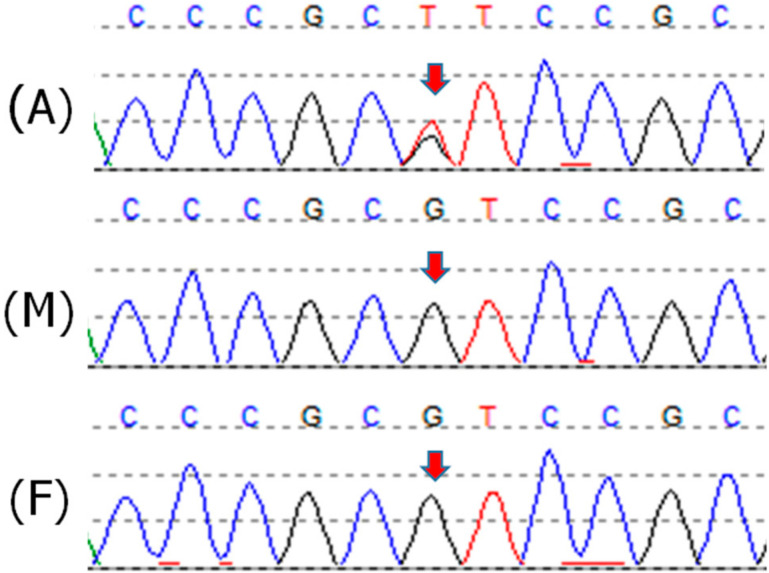
Sanger sequencing electropherograms from Patient 1 (A) and her parents’ blood DNA (M, mother; F, father). The *TP53* exon 5 variant c.469G > T p.(Val157Phe) is indicated by the arrows.

The patient and her family were referred to genetic counseling on the suspect of a germline *TP53* mutation. The family history was uneventful up to the third degree of kinship, and germline testing was offered only based on the peculiar tumor type. The Sanger sequencing analysis of the *TP53* gene, carried out on the proband’s blood DNA, allowed the identification of the c.469G>T p.(Val157Phe) mutation, classified as pathogenic according to the American College of Medical Genetics (ACMG) guidelines [16]. The mutation was absent in blood DNA from both parents, thus, confirming its de novo origin (Figure 1).

Non-paternity was ruled out by performing a short tandem repeats (STRs) segregation analysis, which showed allelic concordance between the patient and her parents. Residual tumor tissue was not available for further analyses, aimed at assessing the presence of the mutation and a putative second mutation/loss of heterozygosity (LOH) at the tumor level.

### 2.2. Patient 2

A 9-year-old boy presented with intracranial hypertension like headache. Due to the onset of vomiting, he was referred to a pediatric hospital for clinical and radiological assessment. Computed tomography (CT) scans and magnetic resonance imaging (MRI) of the brain revealed a mass in the left cerebellar hemisphere, extending across adjacent structures, perilesional edema, and dilated lateral ventricles. No leptomeningeal dissemination was observed on radiological examinations. He underwent a surgical subtotal excision of the neoplasm, with postoperative radiological investigations revealing residual disease at the left middle cerebellar peduncle and suspected residual disease at the cranial margin of the resection site (Figure S3a,b). No disease localization in the spinal cord or CSF was observed.

Centrally reviewed histopathological examination revealed a desmoplastic/nodular medulloblastoma, SHH-activated and p53 overexpressed (Figure S4).

Postoperative recovery was unremarkable, and the patient started a regimen of chemotherapy, with three cycles of etoposide and carboplatin, autologous stem cells harvest after the second cycle, and one cycle of high dose methotrexate and vincristine. He thereafter received two cycles of myeloablative thiotepa with autologous stem cells transplantation. In addition, he was treated with conventional photon craniospinal irradiation (23.4 Gy) with a boost dose to the tumor bed (30.6 Gy), achieving radiologically complete remission. Currently, 16 months after surgery, the patient is in good clinical condition with no evidence of the disease at radiological examinations (Figure S3c).

**Figure 2:**
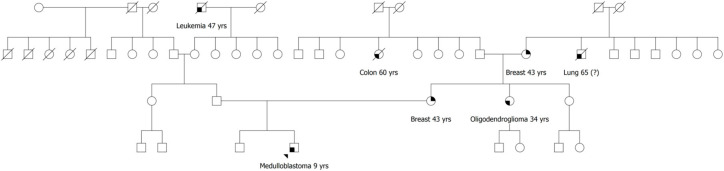
Family pedigree of Patient 2; the proband is indicated by the arrowhead; relatives are included up to the third degree of kinship; oncological diseases and age at onset are reported below each affected individual. Yrs, years; (?), uncertain information.

In consideration of the characteristics of the tumor and its immunohistochemistry staining pattern, the patient was referred to genetic counseling. The pedigree assessment highlighted a family history suggestive of LFS (Figure 2). Due to her personal and family history, positive for premenopausal breast cancer, the patient’s mother had previously undergone a comprehensive *BRCA1*/*BRCA2* molecular analysis, which resulted negative.

**Figure 3:**
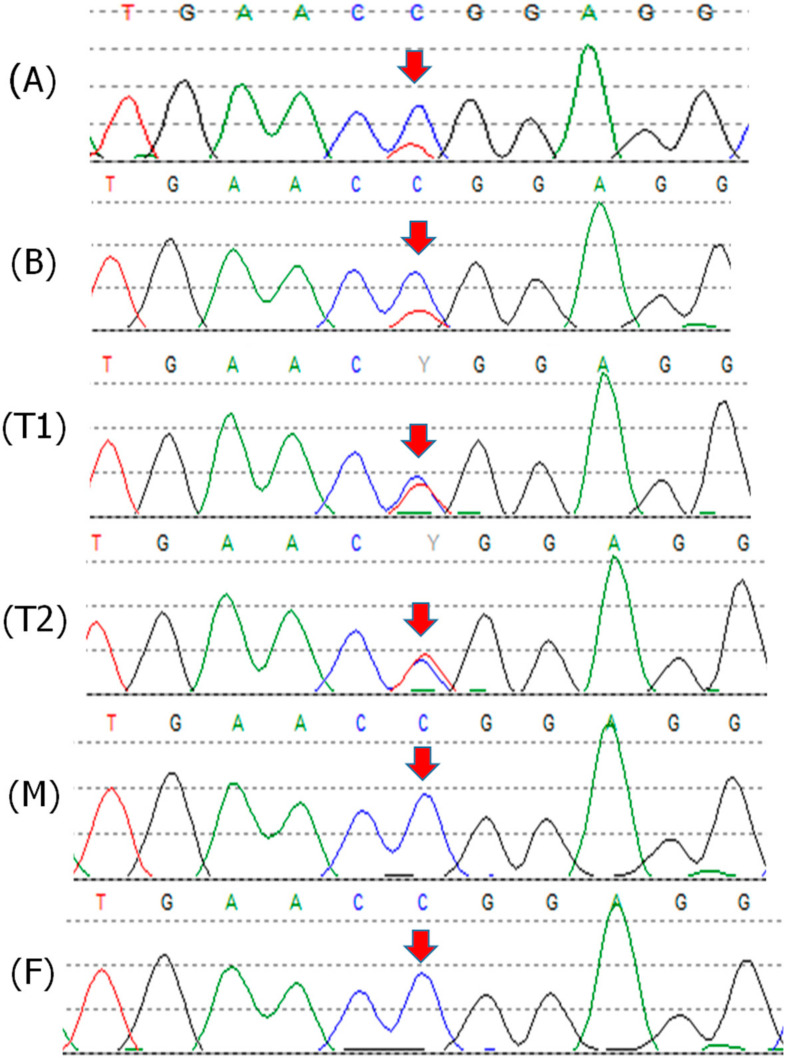
Patient 2 Sanger sequencing electropherograms from blood (**A**), buccal swabs (**B**), tumor tissues (T1, T2), and his parents’ blood DNA (M, mother; F, father). The *TP53* exon 7 variant c.742C>T p.(Arg248Trp) is indicated by the arrows. The quantification of the mutation frequency, by Mutation Surveyor^®^ software (v5.1.0) (SoftGenetics LLC, State College, PA, USA), was as follows: A,B = 25%, T1 = 39%, T2 = 48%, M,F = 0%.

The analysis of the *TP53* gene, carried out through Sanger sequencing on the proband’s blood DNA, allowed the identification of the missense variant in exon 7 c.742C>T p.(Arg248Trp) (Figure 3A). The mutation has been previously reported in LFS families and is considered pathogenic. However, in the proband’s DNA, a difference in the height of the peaks was observed, with the wild-type allele more represented indicating likely somatic mosaicism. The quantification of the allele ratio, performed through Mutation Surveyor software, confirmed a mean allele frequency of the pathogenic variant of about 25% in blood. Subsequent analyses were performed on the proband’s DNA extracted from buccal swabs (Figure 3B) and two tumor specimens (T1 and T2, Figure 3). The average allele frequency of the variant detected in buccal cells was similar to that of leukocyte DNA (i.e., ~25%), indicating that the variant is likely to be present in about half of the cells from two different embryonic germ layers. In both the tumor specimens, the variant was more represented compared with constitutive DNA, with an average allele frequency of 39% in the first analyzed sample (T1) and 48% in the second (T2).

Targeted sequencing on blood DNA from the proband’s parents ruled out the presence of the variant in both the parents, further confirming the hypothesis of a de novo somatic mosaic mutation in the proband. In order to investigate the presence of a different *TP53* mutation in the maternal family, sequencing of the whole *TP53* gene was also performed on blood DNA from the proband’s mother by NGS, with no evidence of either single nucleotide variants or large rearrangements.

**Figure 4:**
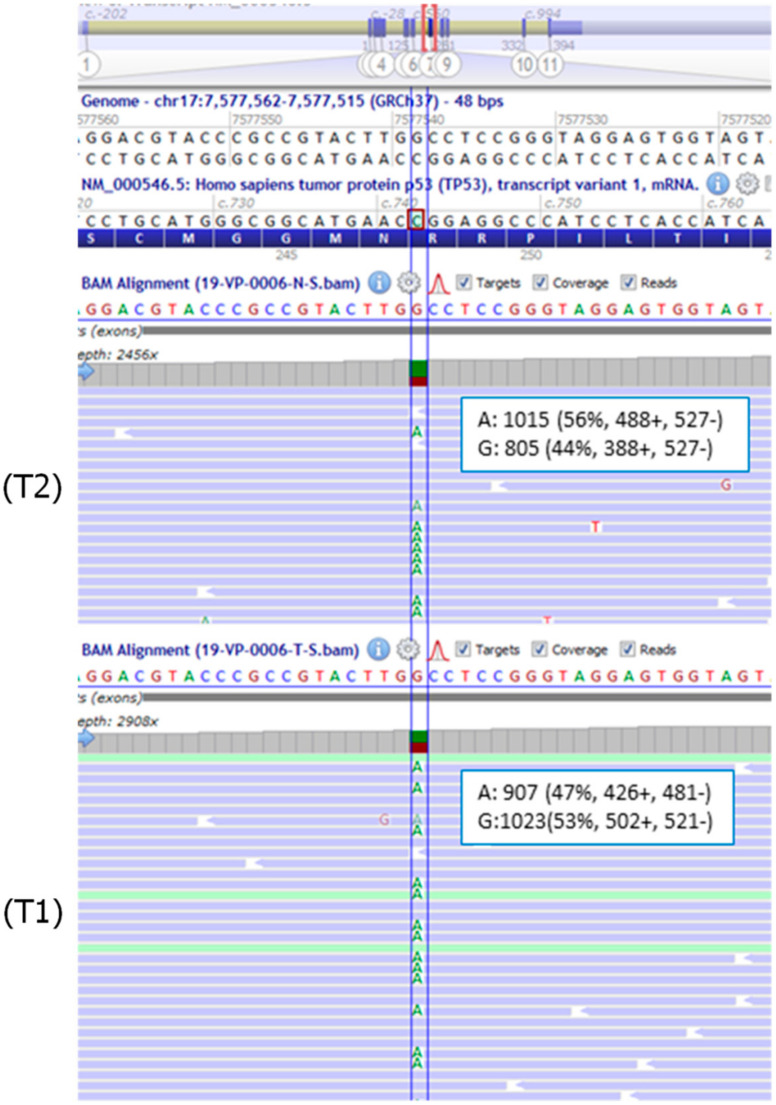
BAM (Binary Alignment Map) files visualization, using Alamut Visual software v.2.15 (Interactive Biosoftware, Rouen, France), of the variant in the two tumor specimens (T1) and (T2) from Patient 2. Partial sequence of *TP53* NM_000546.5 is shown. The position of the *TP53* exon 7 mutation c.742C>T p.(Arg248Trp) is highlighted by the blue line. As *TP53* is located at chromosome 17 on the reverse strand, the non-reference base detected is adenine (A). The frequency and absolute number of reads on each strand are indicated for the mutant and the wild-type alleles.

The NGS analysis with the Oncopan Panel, on both the tumor specimens, revealed the presence of the *TP53* variant c.742C>T p.(Arg248Trp), in 47% of reads (907/1930) in one sample (T1) and 56% (1015/1820) in the other (T2) (Figure 4). The difference in the mutation frequency between the two specimens suggested a higher tumor fraction in sample (T2) compared with sample (T1). No additional pathogenic or likely pathogenic variants of *TP53*, or other genes included in the NGS panel, were identified in both tumor specimens.

**Figure 5:**
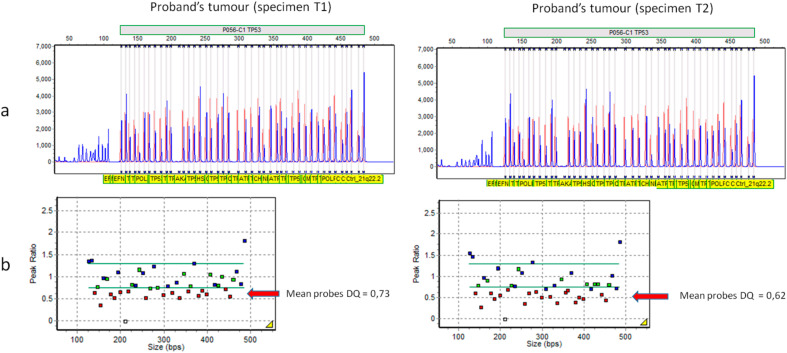
Assessment of loss of heterozygosity (LOH) in the tumor from Patient 2 (specimens T1 and T2) using the SALSA^®^ MLPA^®^ P056-C1 probe mix (MRC Holland, Amsterdam, the Netherlands). (**a**) Raw data indicating a deletion of the *TP53* probes (sized 167, 176, 199, 218, 225, 258, 284, 298, 318, 346, 389, 398, 407, 442, and 452 kb). (**b**) Graphical representation of the results using GeneMarker^®^ software v.2.7.0 (SoftGenetics LLC, State College, PA, USA). The arrows indicate the deleted probes. The expected result for a heterozygous deletion is 0.40 < DQ < 0.65.

Albeit heterogeneous, these specimens appeared to be enriched for the mutation and with a likely loss of heterozygosity (LOH) involving the normal allele. In support of this hypothesis, the analysis of *TP53* single nucleotide polymorphisms (SNPs) (rs1642785, rs1376609066, rs1042522, rs2909430), which were heterozygous at Sanger sequencing in blood DNA, showed an average allele frequency of about 27% in the tumor, indicating LOH in more than 60% of the analyzed cells. In addition, the MLPA analysis confirmed a difference in the extent of the loss between the two tumor specimens, highlighting a higher tumor cellularity in specimen T2 (Figure 5).

In order to assess the allele frequencies with a quantitative method, a qPCR analysis was also performed. This approach confirmed in both blood and buccal cells a mutation frequency of about 26%. The relative quantification was calibrated on the WT allele of normal control samples, including the proband’s mother. The value of 26% was subsequently used as a reference to obtain the corresponding frequency of the variant in the tumor specimens. The results were concordant with the NGS data and showed an average mutation frequency of 42% in sample (T1) and 77% in sample (T2).

## 3. Discussion

The incidence of de novo mutations has recently emerged to be higher than previously expected in several different diseases, with important implications for genetic counseling [17,18,19,20,21,22]. A de novo mutation may occur either at a pre-zygotic stage (e.g., in gametes) or in dividing post-zygotic cells [23]. Recent lines of evidence suggest that the post-zygotic mutation rate per cell division during early embryogenesis might be higher than the average mutation rate in gametes, with about 10% of the de novo mutations observed in children deriving from parental mosaicism occurring during the first cell divisions [24].

In large medulloblastoma cohorts, the role of *TP53* has been extensively investigated, though the prevalence of de novo mutations has not been addressed [10,11,13,25]. In the *TP53* germline mutation database of the International Agency for Research on Cancer (IARC), 45 carriers from 44 different families were affected with medulloblastoma [26]. Information about the mode of inheritance was available for 14 patients, three of whom (21%) harbored de novo mutations. In addition, in one family reported by Renaux-Petel and colleagues, the proband’s mutation was identified at a very low allele frequency (5%) in the healthy father’s blood [4].

In our study, we aimed at characterizing *TP53* mutations and assessing their parental origin in a monocentric cohort of medulloblastoma patients. To avoid overlooking de novo mutations, we considered patients to be offered germline testing based on p53 immunostaining positivity. Although it usually fails to identify truncating mutations, this approach has the advantage of avoiding the typical selection bias of the LFS clinical diagnostic criteria, which take into account family history also.

Among the patients showing tumor p53 overexpression, Patient 1 was the only tumor-affected individual in a large and informative family. The identified c.469G>T p.(Val157Phe) mutation, a rare variant never reported in healthy individuals, was previously described in LFS patients and is considered a hotspot somatic mutation in different types of cancers [26]. In addition, functional studies demonstrated that it determines structural alteration and impaired function of the p53 protein [27,28]. Unfortunately, due to the unavailability of the residual pathologic material, we could not assess the presence of LOH or a second mutation in the tumor.

Post-zygotic mutations of *TP53* have been previously described. However, the number of reported individuals with mosaic mutations is still very limited, with only 10 cases reported to date (Table 1).

The second patient herein described represents the first report of medulloblastoma associated with a *TP53* mosaic mutation. The analysis on blood DNA of the patient revealed the presence of the *TP53* mosaic variant c.742=/C>T p.(Arg248=/Trp) with an estimated allele frequency of about 25%.

The variant is rare in the European population (allele frequency in gnomAD, 0.000008) and functional studies demonstrated that it abolishes DNA-binding and transactivation [32,33,34]. It is considered a hotspot mutation, being the fourth most frequent germline variant, identified in 49 different LFS families as reported in the latest version of the IARC *TP53* database [26,35]. At the somatic level, it is the fifth most common mutation among all human cancers [36]. Moreover, the variant has been identified in two other mosaic carriers, both affected with choroid plexus carcinoma, further confirming its role as an important mutational hotspot (Table 1) [4,31]. Previous studies confirmed a dominant negative effect of the mutation and showed that it is associated with the highest penetrance among all *TP53* hotspot mutations, with 58% of carriers affected by the age of 30 years [1,35]. A remarkable finding in Patient 2 was that the allele frequency of the mutation was about 25% in both blood and buccal swabs, indicating that the mutation did not undergo a positive selection process throughout embryo development. A possible explanation for this phenomenon would be that p53 is not essential for normal embryonic development, as demonstrated in both zebrafish and mouse models [37,38].

Notably, in the IARC database, 33 out of 98 germline carriers of the c.742C>T p.(Arg248Trp) mutation with known tumor type (34%) developed a brain malignancy [26]. Among all mutation carriers, central nervous system manifestations are observed in about 10–15% of cases, with an overall bimodal distribution across the ages [1,39]. The most common histologic types include choroid plexus tumors and medulloblastomas, in infants and children, respectively, and mainly astrocytomas and glioblastomas in adults [40].

Medulloblastoma is the most common malignant brain tumor in children. The 2007 WHO classification of CNS tumors recognizes four histological variants: classic, desmoplastic/nodular, extensive nodularity, and anaplastic/large cell medulloblastoma. In the last decade, the clinical and biological heterogeneity of medulloblastoma has been evaluated in terms of transcriptional and methylation profile with the definition of four distinct molecular subgroups: WNT, Sonic Hedgehog (SHH), group 3, and group 4 [41]. These four groups have different genetic backgrounds, transcriptional profiles, demographics, recurrence patterns, and outcomes [12]. In a large cohort of 1022 patients with medulloblastoma, germline *TP53* mutations were found in 1% of patients, all affected with SHH-medulloblastoma and diagnosed between 5 and 16 years of age. In this subgroup, the reported prevalence of germline *TP53* mutations was 8% [13]. In this light, both the prevalence of germline mutations and the age range of affected germline carriers in our cohort were in line with the data from other cohorts. In Patient 2, although the pathology and clinical features were consistent with a pathogenetic role of the identified mosaic mutation, we carried out further analyses aimed at demonstrating its driver role. Two different FFPE tumor specimens were analyzed with different molecular techniques, which consistently evidenced a variant frequency higher than that observed in blood or buccal cells. Nevertheless, due to the different sensitivity of the methods, Sanger sequencing, NGS, and qPCR provided slightly different results, with mutation frequencies ranging from 39% to 47% in specimen (T1) and from 48% to 77% in specimen (T2). The difference in the allele frequency between the two specimens was likely due to both different tumor cellularity, which ranged 50–70%, and the presence of a variable fraction of normal wild-type cells. Unfortunately, a precise selection of tumor cells was difficult due to the limited available tissue. Sampling was carried out through coring of the FFPE block, presumably leading to the inclusion of surrounding normal tissue. However, the markedly higher allele frequency of the mutation, along with the observed LOH in the tumor specimens, suggested that the tumor originated from hemizygous mutated cells. Taken together, these data point out that the mosaic variant identified in Patient 2 is likely to be a driver mutation in tumor development.

A few other interesting points concerning the case of Patient 2 should be highlighted. First, surprisingly, the maternal family history was highly suggestive of LFS (Figure 2) [1]. Notwithstanding, his mother, affected with breast cancer at age 43 years, resulted negative at both *BRCA1*/2 and *TP53* testing. These findings, thus, emphasize that patients with SHH-type medulloblastoma showing p53 overexpression/mutation should be always considered for *TP53* germline testing, regardless of family history.

Another aspect, which represents a relevant yet unanswered question, is whether a mosaic *TP53* mutation might be responsible for a milder LFS phenotype. As concerns the penetrance, interestingly, all mosaic cases, some of whom with a mutant allele frequency in blood below 10%, were affected with typical tumors in the spectrum of LFS both for type and age at onset. A possible explanation would be that even a small fraction of cells harboring these mutations is sufficient to confer a high risk of developing cancer. However, in this scenario, the estimate of the risk would still depend on the actual presence of the mutation in a specific tissue. Conversely, the hypothesis of a high penetrance in mosaic carriers might be burdened by an ascertainment bias, since *TP53* testing is usually offered to patients fulfilling the stringent LFS clinical diagnostic criteria. In both cases, the real penetrance might be lower compared with conventional LFS patients, with potential important implications for surveillance.

Additionally, disease severity and clinical outcome could be possibly influenced by somatic mosaicism. Approximately 30% of medulloblastomas are classified as SHH tumors. Outcomes in this group vary according to clinical (age, metastatic status) and molecular (*MYCN* amplification, *TP53* mutation status) characteristics. Patients with SHH-medulloblastoma rarely have a disseminated tumor at diagnosis and the prognosis is intermediate, with a 5-year overall survival of approximately 75% when treated with standard therapy [41]. Patients with SHH-medulloblastoma and *TP53* mutation represent a small subgroup, with an annual accrual rate of approximately 5–10 patients in Europe. Although young children have a more favorable outcome, patients with *TP53*-mutated SHH medulloblastoma do poorly, with a 5-year overall survival of 27% [10,13,42]. Notably, in other tumor types, the mutation identified in Patient 2 has been associated with a worse prognosis compared with other *TP53* mutations, as observed in a large cohort of women affected with breast cancer [43]. Although the follow-up time is very short, with respect to Patient 1 and other medulloblastoma-affected LFS patients reported in the literature, who showed a rapidly progressive clinical course, Patient 2 has been in complete remission for more than one year after the start of treatment. It might be speculated that the presence of a lower fraction of mutated cells, in the context of a tumor-affected tissue, could foster a better response to therapies and reduce the development of therapy-induced cancer cells. This hypothesis is in line with the observation that early acquisition of *TP53* mutations in clonal hematopoiesis events contributes to the poor responses to chemotherapy in patients with hematological tumors [44]. However, additional studies are necessary in order to investigate a putative favorable effect of constitutional mosaicism, compared with inherited mutations or pre-zygotic de novo mutations, on tumor prognosis and treatment outcome.

## 4. Materials and Methods

### 4.1. Patients

Between January 2015 and June 2020, 24 medulloblastoma samples from patients followed at Fondazione IRCCS Istituto Nazionale dei Tumori underwent a centrally reviewed histopathological examination, at the Department of Human Neurosciences, Sapienza University of Rome, and resulted SHH-activated. The molecular subgroup was evaluated through the expression analysis of four immunohistochemical markers: GAB1, b-catenin, filamin A, and YAP1, as previously described [45]. Among this cohort of patients, only those with tumors showing an intense p53 staining pattern were selected for the study and referred to genetic counseling. Of the three identified cases, only two provided informed consent for further molecular analysis.

### 4.2. DNA Samples

Informed consent, for the collection of biological specimens and subsequent molecular analyses for the identification of germline variants in tumor-predisposing genes, was obtained from the parents of both probands. Peripheral blood samples were collected from the probands and their parents. Buccal epithelial cells were collected from Patient 2 by mouth swab.

Whole blood DNA and genomic DNA from buccal epithelial cells was isolated through the MagCore^®^ Super automatic workstation with the MagCore^®^ Genomic DNA Whole Blood Kit (Diatech LabLine SRL, Jesi, Italy).

Two FFPE samples of the tumor developed by Patient 2, both with an estimated tumor cellularity of about 60%, were also retrieved for DNA extraction. A 1.0 mm core needle was used to sample the tissue block in tumor foci to allow for positive enrichment of tumor cells. Hematoxylin/Eosin (Diapath) staining was performed according to standard protocol before and after the isolation of selected areas. Tumor DNA was extracted through the Gene Read DNA FFPE kit (Qiagen, Hilden, Germany) according to the protocol instructions.

### 4.3. Immunohistochemistry

Immunohistochemical analyses were performed on an automated-stainer (Leica Bond III) on freshly cut 3-μm FFPE slides. Samples were scored on the scale 0–3 and were quantified by counting at least 1000 tumor cells; staining intensity was scored as “no expression” (0), “weak expression” (1+), “moderate expression” (2+), and “strong expression” (3+). P53 (DO-7 Leica) was scored as overexpressed when neoplastic cell nuclei showed staining intensity >2, compared to endothelial cell nuclei that served as internal control. Samples were scored as positive for p53 overexpression when >60% of neoplastic cells showed strong nuclear expression (>2) associated with the negativity of internal control. The p53 staining was assessed across the entire tumor section, blind to other pathological and biomarker data and *TP53* mutational status.

### 4.4. STRs Segregation Analysis

Short tandem repeats (STRs) segregation analysis was carried out on blood DNA from both patients and their parents using the GenePrint^®^ 10 System Panel (Promega, Madison, WI, USA, which allows the detection of the following 10 loci: TH01, TPOX, vWA, Amelogenin, CSF1PO, D16S539, D7S820, D13S317, D5S818, and D21S11. The multiplex PCR were done according to the manufacturer’s instructions and the reaction products were run on the 3730xl DNA analyzer (Thermo Fisher Scientific, Waltham, MA, USA. The electropherograms were analyzed with the Gene Marker Software v.2.7.0 (SoftGenetics, State College, PA, USA).

### 4.5. Sanger Sequencing

*TP53* coding exons (2–11) and flanking regions were simultaneously amplified at the annealing temperature of 60 °C, with the AmpliTaq Gold kit (Applied Biosystems; Thermo Fisher Scientific, Inc., Waltham, MA, USA). Sequencing was performed on purified PCR products by using the BigDye^®^ Terminator v.3.1 Cycle Sequencing kit (Thermo Fisher Scientific, Inc.) and run on the 3500 Dx Genetic Analyzer (Thermo Fisher Scientific, Waltham, MA, USA) after purification with Agencourt CleanSeq^®^—Beckman Coulter. Sequences were analyzed by Mutation Surveyor^®^ Software v5.1.0 (SoftGenetics, LLC, State College, PA, USA). The analysis was carried out on all available tissues from Patient 2 (tumor, blood, buccal cells) and on blood DNA from Patient 1.

### 4.6. Multiplex Ligation-Dependent Probe Amplification (MLPA)

Analysis of large deletions and duplications of *TP53* was carried out, in the probands’ blood DNA and in the tumor DNA from Patient 2 with the *TP53* SALSA MLPA KIT-P056 (C1) probemix (MRC-Holland, Amsterdam, the Netherlands), following the manufacturer’s instructions. MLPA products were run on the 3730Xl DNA Analyzer (Applied Biosystems; Thermo Fisher Scientific, Inc.) with the Gene Mapper Module (Applied Biosystems; Thermo Fisher Scientific, Inc.). The results were analyzed through the Gene Marker Software v2.7.0 (SoftGenetics, LLC, State College, PA, USA).

### 4.7. Next Generation Sequencing (NGS)

About 150–200 ng of dsDNA, according to Qubit dsDNA HS assay kits fluorometric quantification, were sheared by the Sure Select Enzymatic Fragmentation kit (Agilent Technologies Inc., Santa Clara, CA, USA). The NGS library was created using Sure Select XT2 Low input Custom library probes (Agilent Technologies Inc.), and sequencing was performed on MiSeq (Illumina Inc., San Diego, CA, USA) using 2 × 150 bp paired-end sequencing. Data collection was performed with the MiSeq Reporter (MSR) software v.2.6.2.3, using the ‘FastQ only’ workflow. The run quality was evaluated by Illumina Sequencing Analysis Viewer v.1.9.1, while the Bioinformatics pipeline for the annotation of the vcf files was developed in-house, in collaboration with enGenome Software Company (Pavia, Italy). Paired-end reads were mapped on the Human hg19 genome. Full coverage of the coding regions, mean read depth of 650× and read coverage of >50× were obtained.

The NGS custom panel, named Oncopan, can detect Single Nucleotide Variants (SNV) and small ins/del of the following genes: *APC*, *ATM*, *BARD1*, *BMPR1A*, *BRCA1*, *BRCA2*, *BRIP1*, *CDH1*, *CDKN2A* (α e β), *CDK4* (exon 2), *CHEK2*, *CTNNA1*, *EPCAM*, *FANCM*, *MLH1*, *MSH2*, *MSH3*, *MSH6*, *MUTYH*, *NBN*, *NHTL1*, *PALB2*, *PMS2*, *POLD1*, *POLE*, *PTEN*, *RAD51C*, *RAD51D*, *SMAD4*, *STK11*, *TP53*, *KRAS*, *NRAS*, *BRAF*, *EGFR*, *HER2*(*ERBB2*), *PIK3CA*. The analysis was carried out on tumor samples from Patient 2.

### 4.8. Quantitative Polymerase Chain Reaction (qPCR)

An amount of 20 ng of DNA was amplified in a reaction volume of 10 μL, containing the following reagents: 5 μL of TaqMan Fast Advanced MasterMix 2x (Thermo Fisher, Foster City, CA, USA), 0.25 μL of Custom TaqMan SNP assay 40× (Thermo Fisher, Foster City, CA, USA). Real-time PCR was carried out on the 7500 real-time PCR system (Applied Biosystems, Foster City, CA, USA), using a pre-PCR step of 20 s at 95 °C, followed by 40 cycles of 3 s at 95 °C and 30 s at 60 °C. The primer and probe sequences were the following: *TP53* Exon 7-Forward primer 5′-AACTACATGTGTAACAGTTCCTGCAT-3′, Reverse primer 5′-GAGTCTTCCAGTGTGATGATGGT-3′; probe 1 (VIC) 5′-TGGGCCTCCGGTTCA-3′, probe 2 (FAM) 5′-TGGGCCTCCAGTTCA-3′. The analysis was carried out on all available tissues from Patient 2 (tumor, blood, buccal cells).

## 5. Conclusions

This study highlighted the occurrence of TP53 de novo mutations in medulloblastoma patients, pointing out that germline testing should be offered to patients with SHH-type medulloblastoma and p53 overexpression, regardless of family history. Moreover, we described the first patient reported to date with medulloblastoma and a constitutional TP53 mosaic mutation. The molecular analyses on multiple tissues confirmed that the mutation occurred at an early stage of embryogenesis and indicated a driver role in tumor development. Compared with other medulloblastoma-affected LFS individuals, the clinical course seemed to be milder, although longer follow-up time and data on additional cases are needed to investigate a putative favorable effect of mosaicism on disease severity.

## Figures and Tables

**Table 1:** Li-Fraumeni syndrome patients with *TP53* mosaic mutations.

Ref.	Gender	Tumor (Age)	*TP53* Variant	Allele Fraction in Blood	Allele Fraction in Other Tissues	LOH
Patient 2	M	MB (9)	c.742=/C>T p.(Arg248=/Trp)	26%	26% (BS); 42–77% (T)	yes
Prochazkova et al., 2008 [29]	F	ACA (1); OS (5)	c.844=/C>T p.(Arg282=/Trp)	33% *	33% * (U); 50% * (BS)	N/A
Behjati et al., 2014 [30]	M	MFB (0, 7); sarcoma (0, 9); NB (1, 3)	c.743=/G>A p.(Arg248=/Gln)	14%	86–97% (T)	yes
Trubicka et al., 2017 [31]	M	CPC (1, 5)	c.742=/C>T p.(Arg248=/Trp)	13%	33% (BS); 15% (U); 87.5% (T)	yes
Renaux-Petel et al., 2018 [4]	M	ACC (0, 3)	c.722=/C>T p.(Ser241=/Phe)	17%	NA	yes
	F	ACC (0, 7)	c.548=/C>A p.(Ser183=/ *)	17%	NA	yes
	F	ACC (1)	c.75-10_81=/dup p.(Glu28=/Cysfs * 22)	4%	NA	N/A
	M	CPC (2)	c.742=/C>T p.(Arg248=/Trp)	14%	NA	N/A
	M	Atypical CPP (0, 5)	c.818=/G>A p.(Arg273=/His)	19%	NA	N/A
	F	BBC (27, 34)	c.1024=/C>T p.(Arg342=/ *)	17%	NA	N/A
	F	OS (12); BBC (35, 35)	c.375+1=/G>A p.(=/?)	7%	NA	yes

Age is reported in years; the hotspot c.742=/C>T p.(Arg248=/Trp) variant is highlighted in bold. LOH—loss of heterozygosity; MB—medulloblastoma; ACA—adrenocortical adenoma; OS—osteosarcoma; MFB—myofibroblastoma; NB—neuroblastoma; CPC—choroid plexus carcinoma; CPP—choroid plexus papilloma; ACC—adrenocortical carcinoma; BBC—bilateral breast cancer; BS—buccal swab; U—urine; T—tumor; N/A—not available. * data derived only from Sanger sequencing and Denaturing Gradient Gel Electrophoresis (DGGE).

## Supplementary Materials

The following are available online at https://www.mdpi.com/2072-6694/12/9/2503/s1, Figure S1: Brain magnetic resonance images of Patient 1, Figure S2: IHC images of Patient 1, Figure S3: Brain magnetic resonance images of Patient 2, Figure S4: IHC images of Patient 2.
