# Supplementary material for: Pre- and Post-Zygotic TP53 De Novo Mutations in SHH-Medulloblastoma
Source: Cancers (Basel). 2020 Sep 3;12(9):2503. doi: 10.3390/cancers12092503 (PMC7564492; doi:10.3390/cancers12092503)
Supplement: Supplementary file 1 [file cancers-12-02503-s001.pdf]

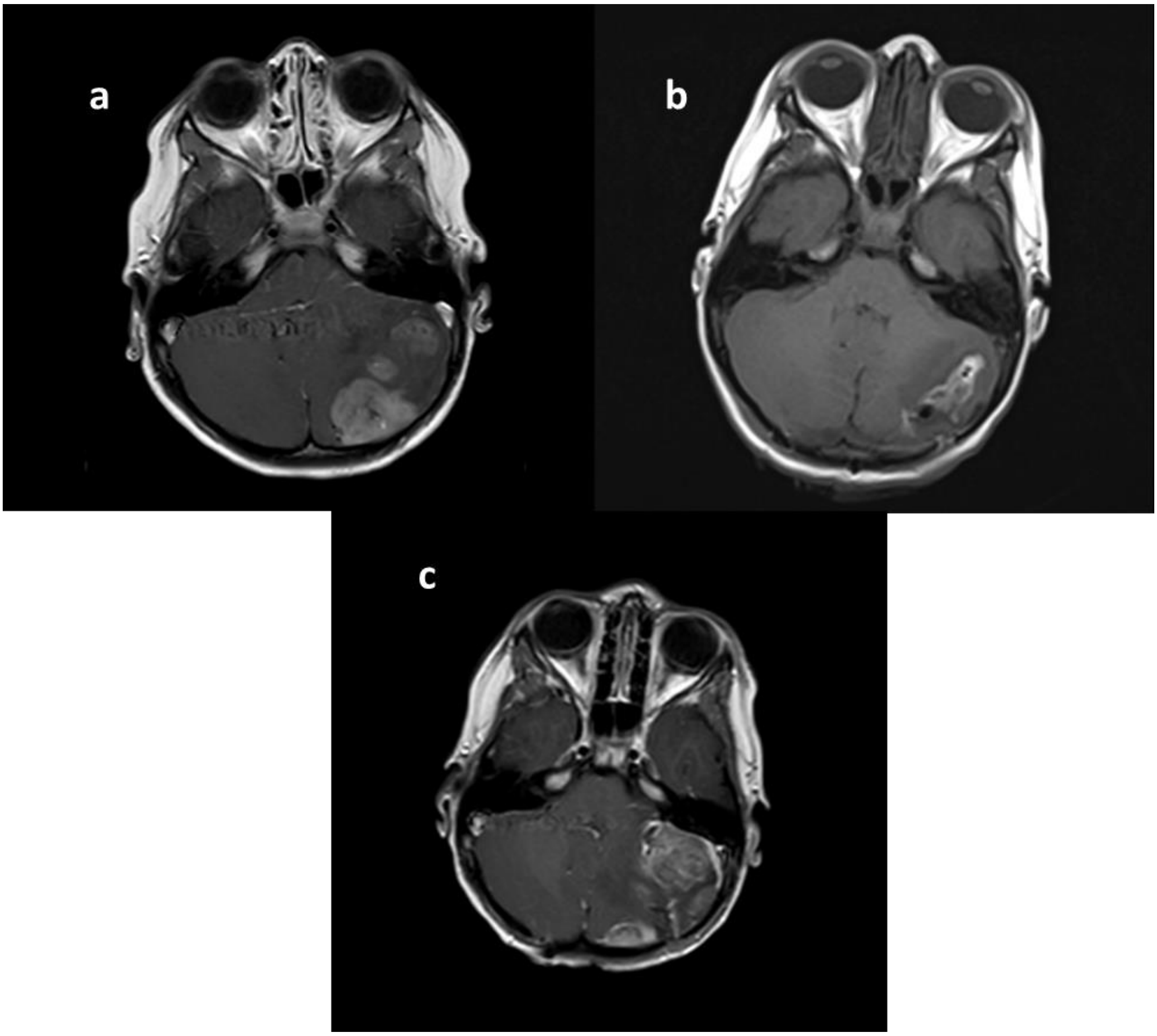

Figure S1. Brain magnetic resonance images of Patient 1 showing the mass in the left cerebellar hemisphere before (**a**) and after (**b**) the surgical subtotal excision; progressive disease 11 months after surgery (**c**).

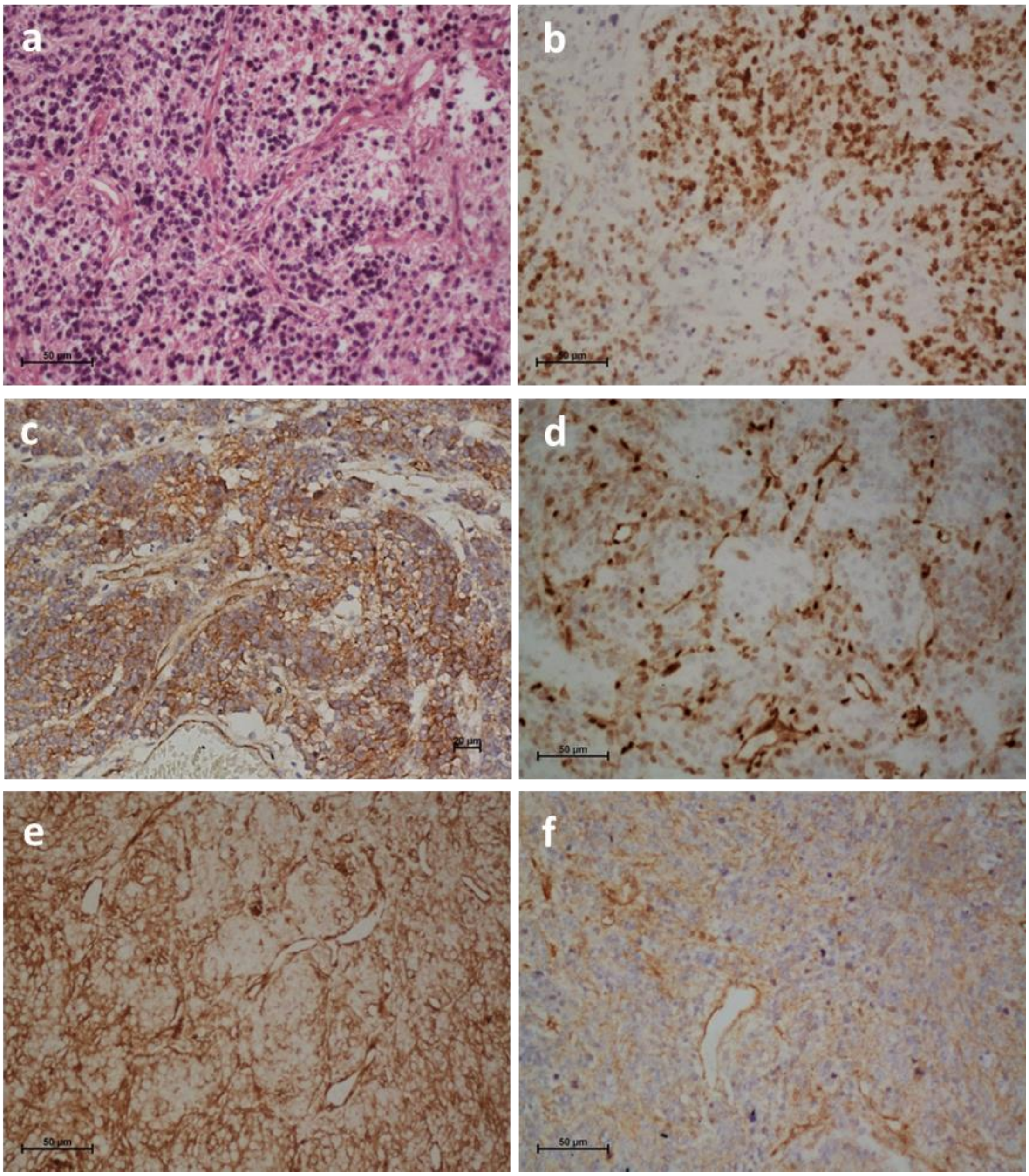

Figure S2. IHC images of Patient 1. H&E staining showing a nodular pattern (**a**); intense diffuse nuclear expression of p53 (**b**); positive staining for GAB1 (**c**), YAP1 (**d**), and Filamin A (**e**); absence of nuclear staining for beta-catenin (**f**).

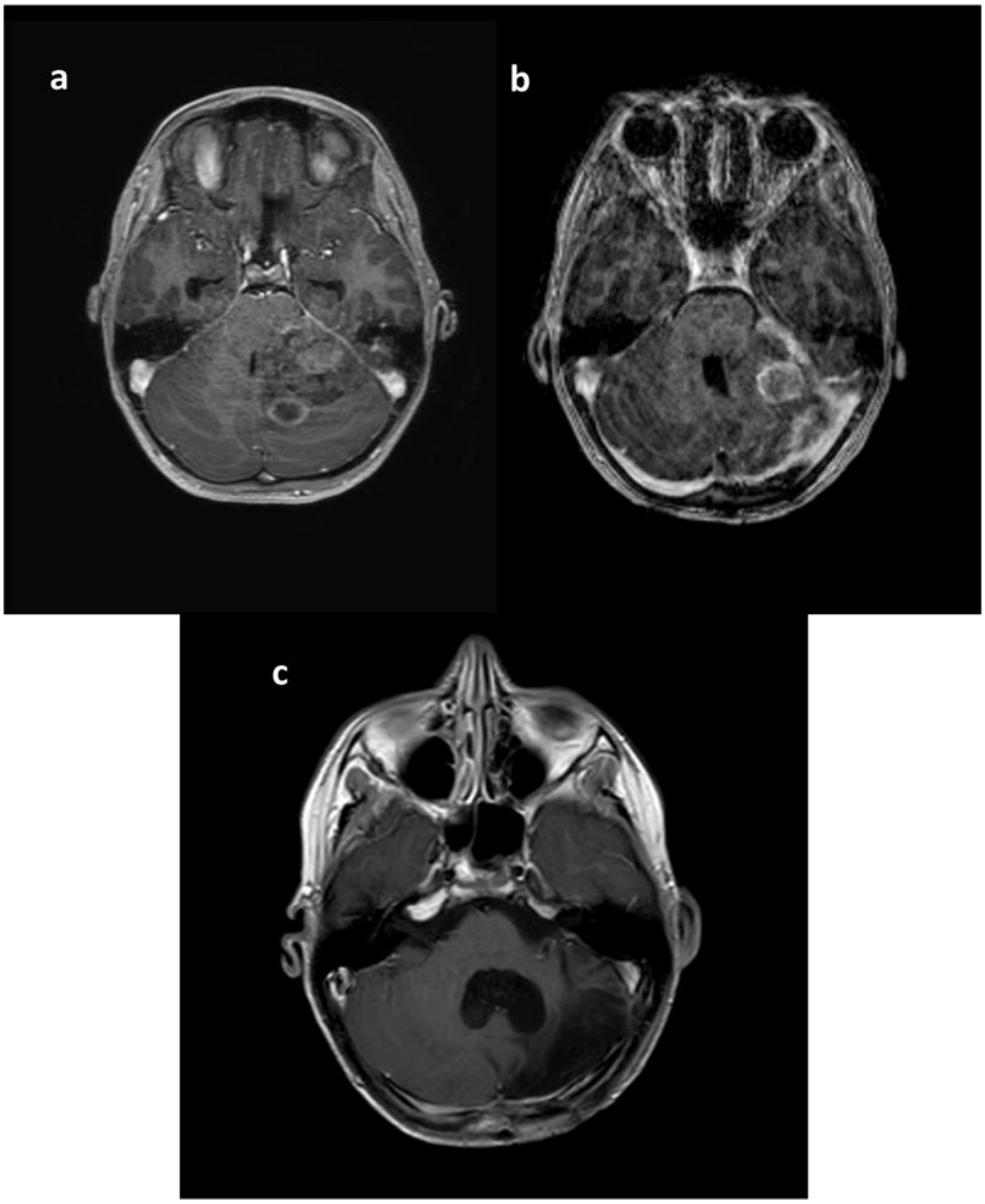

Figure S3. Brain magnetic resonance images of Patient 2 showing the mass in the left cerebellar hemisphere before (**a**) and after the surgical subtotal excision (**b**); no residual disease was observed 16 months after surgery (**c**).

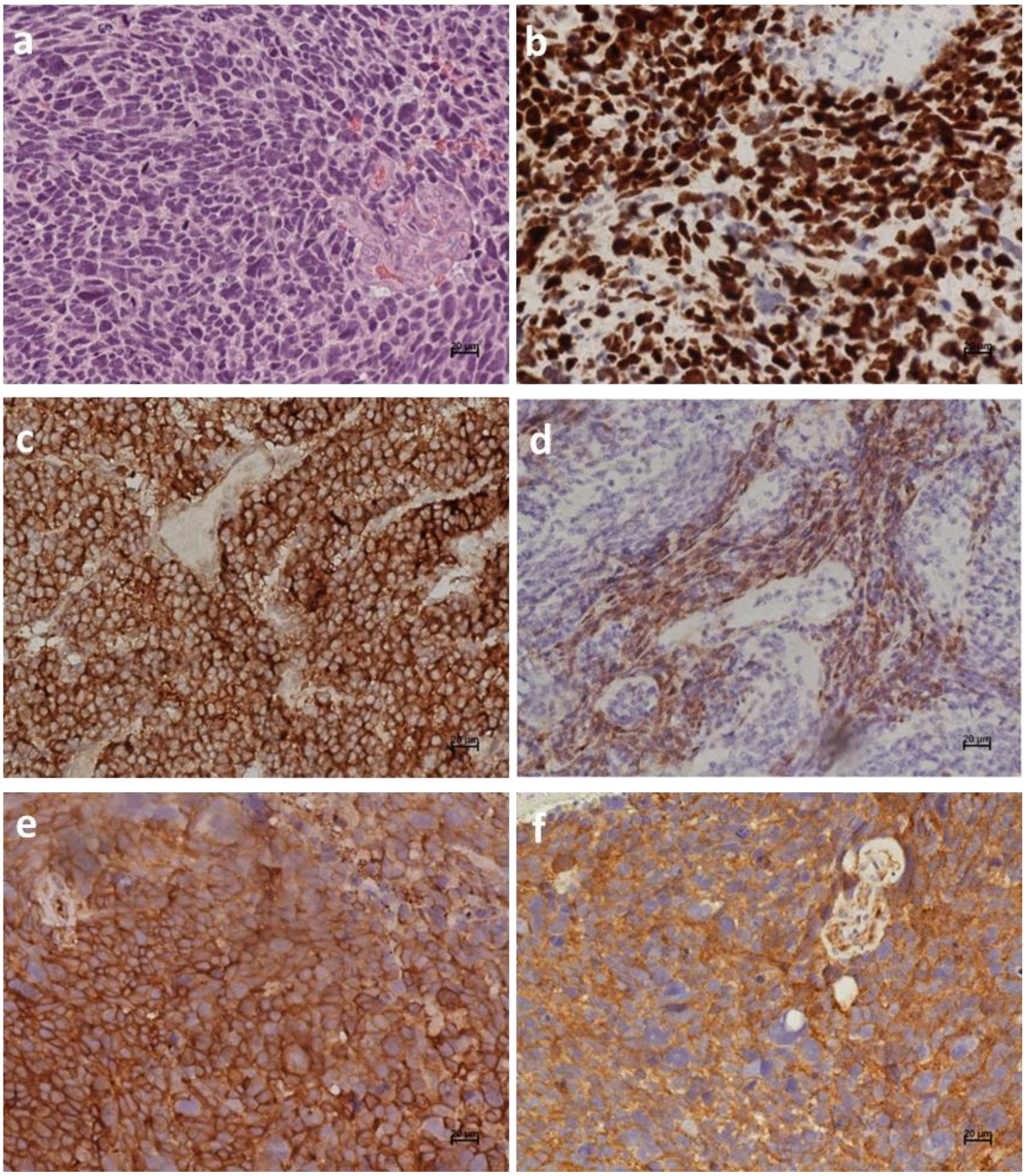

Figure S4. IHC images of Patient 2. H&E staining showing nodules composed of monomorphous cells with neurocytic differentiation, inter-nodular areas showed frank anaplasia with larger cells, marked nuclear pleomorphism and atypical mitoses (**a**); intense diffuse nuclear expression of p53 in >60% of tumor cells (**b**); positive staining for GAB1 (**c**), YAP1 (**d**), and Filamin A (**e**); absence of nuclear staining for beta-catenin (**f**).
